# EBV antibody and gastric cancer risk: a population-based nested case-control study in southern China

**DOI:** 10.1186/s12885-023-10994-0

**Published:** 2023-06-08

**Authors:** Yun Du, Xia Yu, Ellen T. Chang, Li Yin, Shifeng Lian, Biaohua Wu, Fugui Li, Zhiheng Liang, Yumei Zeng, Bing Chu, Kuangrong Wei, Jiyun Zhan, Xuejun Liang, Weimin Ye, Mingfang Ji

**Affiliations:** 1 Zhongshan City People’s Hospital, Cancer Research Institute of Zhongshan City, Zhongshan, 528400 People’s Republic of China; 2 Department of Medical Epidemiology and Biostatistics, Karolinska Institutet, Stockholm, 17177 Sweden; 3 Center for Health Sciences, Exponent, Inc, Menlo Park, CA 94025 USA; 4 Unit of Integrative Epidemiology, Institute of Environmental Medicine, Karolinska Institutet, Stockholm, 17177 Sweden; 5 Department of Pathology, Zhongshan City People’s Hospital, Zhongshan, 528400 People’s Republic of China; 6 Xiaolan Public Health Service Center, Zhongshan, 528400 People’s Republic of China

**Keywords:** Gastric cancer, EBNA1-IgA, VCA-IgA, Nested case-control study, Population-based

## Abstract

**Background:**

We aim to clarify the controversial associations between EBV-related antibodies and gastric cancer risk.

**Methods:**

We analysed the associations between serological Epstein-Barr nuclear antigen 1 immunoglobulin A (EBNA1-IgA) and viral capsid antigen immunoglobulin A (VCA-IgA) by enzyme-linked immunosorbent assay and the risk of gastric cancer in a nested case-control study originated from a population-based nasopharyngeal carcinoma (NPC) screening cohort in Zhongshan, a city of southern China, including 18 gastric cancer cases and 444 controls. Conditional logistic regression was used to calculate the odds ratios (ORs) and corresponding 95% confidence intervals (CIs).

**Results:**

All the sera of cases were sampled before diagnosis and the median time interval was 3.04 (range: 0.04, 7.59) years. Both increased relative optical density (rOD) values of EBNA1-IgA and VCA-IgA were associated with higher risks of gastric cancer with age adjusted ORs of 1.99 (95%CI: 1.07, 3.70) and 2.64 (95%CI: 1.33, 5.23), respectively. Each participant was further classified as high or medium/low risk based on a combination of two anti-EBV antibody levels. Participants in the high-risk group had substantially higher odds of developing gastric cancer than that in the medium/low risk group with an age adjusted OR of 6.53 (95%CI: 1.69, 25.26).

**Conclusions:**

Our research reveals positive associations between EBNA1-IgA and VCA-IgA and gastric cancer risk in southern China. We thus postulate that EBNA1-IgA and VCA-IgA might appear to be potential biomarkers for gastric cancer. More research to further validate the results among diverse populations and investigate its underlying biological mechanism is needed.

**Supplementary Information:**

The online version contains supplementary material available at 10.1186/s12885-023-10994-0.

## Background

In 2020, gastric cancer ranked fifth for cancer incidence and fourth for cancer mortality globally and one in every 13 deaths was due to gastric cancer [1]. The incidence rate in males is two-fold that in females. The age-standardized rate (ASR) among males reached 32.5/100, 000 person-years in Eastern Asia, followed by Eastern Europe of 17.4/100,000. In China, gastric cancer incidence ranked fourth with 20.6/100,000 ASR and its mortality sat third with 15.9/100,000 ASR among all cancer [2]. In Zhongshan City, a southern city in China, gastric cancer ASR was lower than the nationwide level with an incidence of 9.0/100,000 among males and 4.3/100,000 among females [3].

Chronic *H. pylori* infection is the leading cause, accounting for 75% of non-cardia gastric cancer [4]. Besides *H*. *pylori*, other risk factors include tobacco smoking, consumption of preserved food, etc. [1]. About 10% of gastric cancer cases are Epstein-Barr virus (EBV)-positive, using EBV-encoded RNA in-situ hybridization as the gold standard [5]. EBV is the first human tumor-related virus discovered in 1964 [6]. In 1992, EBV particle was detected in the tumor cells of gastric adenocarcinoma [7]. Afterwards, EBV-related gastric cancer was defined as one of the four molecular subtypes of gastric cancer in 2014 [8]. EBV belongs to the human herpes virus with a double-strand DNA genome. It can infect both B cells and epithelial cells. Subsequently, it enters either latent or lytic phase. In the latent phase, EBV could keep long-term infection, interact with the host cell, cause oncogenic events, and promote cancer development eventually, while in the lytic phase, EBV could replicate and be secreted into the blood or tissue and find new host cells, or enter saliva and transmit to other hosts. During the life cycle described above, the virus could transcript some specific genomic loci and translate into proteins to play essential roles in transforming normal cells and preventing apoptosis [9]. At the same time, the human immune response could be activated. Our humoral immune response could produce antibodies. Plasma cells, which are differentiated from B cells, produce three main kinds of immunoglobulin isotypes: IgM, IgG, and IgA. Of these isotypes, IgM is the first to appear when an infection begins and lasts for four to six weeks. IgG increases to a peak after two weeks since infection and remains at a low concentration for the rest of the individual’s life. IgA plays an essential role in the immune function of mucous. Secretory IgA binds to the mucus layer covering the epithelial cells, while serum IgA initiates inflammatory reactions.

These antibodies could be used as biomarkers for cancer screening, diagnosis, and prognosis monitoring. In NPC, EBV-related antibodies have been widely used for cancer screening in high-risk areas with high sensitivity and specificity to improve the early diagnosis [10, 11]. Its utilization, however, for gastric cancer is rare.

To explore the potential utilization of EBV-related antibodies in gastric cancer screening and diagnosis, we analysed the association between Epstein-Barr nuclear antigen 1 immunoglobulin A (EBNA1-IgA) and viral capsid antigen immunoglobulin A (VCA-IgA) in sera and the risk of gastric cancer in a population-based nested case-control study in Zhongshan, a city of southern China.

## Methods

### Study population

The study design is a population-based, nested case-control study in a cohort of 48,171 people participating in NPC screening in three towns (Xiaolan, Minzhong and Gangkou) in Zhongshan, China between 2009 and 2014 (Xiaolan), 2012–2018 (Minzhong) and 2014 (Gangkou). After excluding participants whose ages were not between 30 and 59 (n = 8,326), participants with missing the date of recruitment (n = 4,935), duplicated participants (n = 600), and participants with missing and unreasonable values (n = 81), 39,242 participants were left in the screening cohort. We identified 19 gastric cancer cases until December 31st 2019 through linkage to cancer registry and mortality registry of Zhongshan. We reviewed medical records and pathological reports to confirm the final diagnosis. One gastric stromal tumor case was excluded, leaving 18 gastric cancer cases in final analysis. The date of diagnosis was used as the index date for participants.

We selected 30 controls randomly from the same screening cohort for each case by incidence density sampling by sex, age (in the same age category: 30 ~ 39, 40 ~ 49 and 50 ~ 59), date of initial screening, and the residence town. Controls were alive, not migrating out of Zhongshan city and without history of gastric cancer to the diagnosis date of the matched cases. The date of the corresponding cases was used as the index date for the controls.

### Exposures

Exposures were EBV-related antibodies. Each participant donated 6 mL peripheral whole blood samples at enrollment and during cohort follow-up. Only the initial (earliest) blood sample was used for the present analysis. EBNA1-IgA (Zhongshan Bio-Tech Company, Zhongshan, China) and VCA-IgA (UROIMMUN AG, Lübeck, Germany) were measured in sera using enzyme-linked immunosorbent assay (ELISA), reported with relative optical density (rOD) values. For analysis, the antibody in sera was categorized as positive (rOD ≥ 1) or negative (rOD < 1) according to the manufacturers’ instructions. Combined serologic patterns of VCA-IgA and EBNA1-IgA were classified as high-, medium-, or low-risk according to NPC screening strategy. The detailed procedure was described in our previous publication [10]. Briefly, we used a logistic regression model to identify an optimal combination of EBNA1-IgA and VCA-IgA to discriminate NPC from controls. The sensitivity, specificity, and area under the receiver operating characteristic curve of the combination of EBNA1-IgA and VCA-IgA for predicting NPC were used to develop the following prediction formula: logit P = − 3.934 + 2.203 × VCA-IgA + 4.797 × EBNA1-IgA. We used minimally acceptable false-positive rates of 3% and 7% to define cutoff values for high risk and medium risk (with corresponding logistic regression P = 0.98 and 0.65, respectively).

### Statistical analysis

We used boxplots to visualize the distribution of rOD of EBNA1-IgA and VCA-IgA in gastric cancer cases and controls. Differences in rOD distribution were compared using the Wilcoxon rank-sum test. For the analysis of matched case-control sets, we used conditional logistic regression to calculate odds ratios (ORs) with 95% confidence intervals (CIs) for associations between pre-diagnostic positivity for EBNA1-IgA, VCA-IgA or a combination of the two antibodies and gastric cancer risk, with or without additional adjustment for age at enrollment as a continuous variable. We explored the non-linear associations between rOD of EBNA1-IgA and VCA-IgA and gastric cancer risk by restricted cubic spline with four knots (i.e., knots locations: 0.05, 0.35, 0.65 and 0.95). In light of the incidence density risk-set sampling design, ORs can be interpreted as rate ratios.

### Sensitivity analysis

(1) To evaluate the robustness of the results, we re-performed incidence density sampling 30 times from the screening cohort and calculated the mean of the 30 corresponding adjusted ORs, along with 95% CIs. (2) To exclude an effect of prodromal gastric cancer on anti-EBV antibody levels, we also conducted a sensitivity analysis, restricted to cases with sera samples collected at least two years before diagnosis and their matched controls. 3)We also classified the participants as double-negative, single positive and double-positive groups, and calculated the ORs. 4) To evaluate potential associations between time to gastric cancer onset and the magnitude of VCA-IgA or EBNA1-IgA, we used non-parametric bootstrapping technique (3000 replicates) to obtain the slope and its 95% CI of rOD (i.e., a proxy of magnitude of VCA-IgA/EBNA1-IgA) along years before diagnosis.

All statistical tests were two-sided. We used R (version 4.0.3) for data management and statistical analysis.

### Patient and public involvement

Patients or the public were not involved in the design, conduct, reporting, or dissemination plans of our research.

## Results

### Study population characteristics

**Fig. 1:**
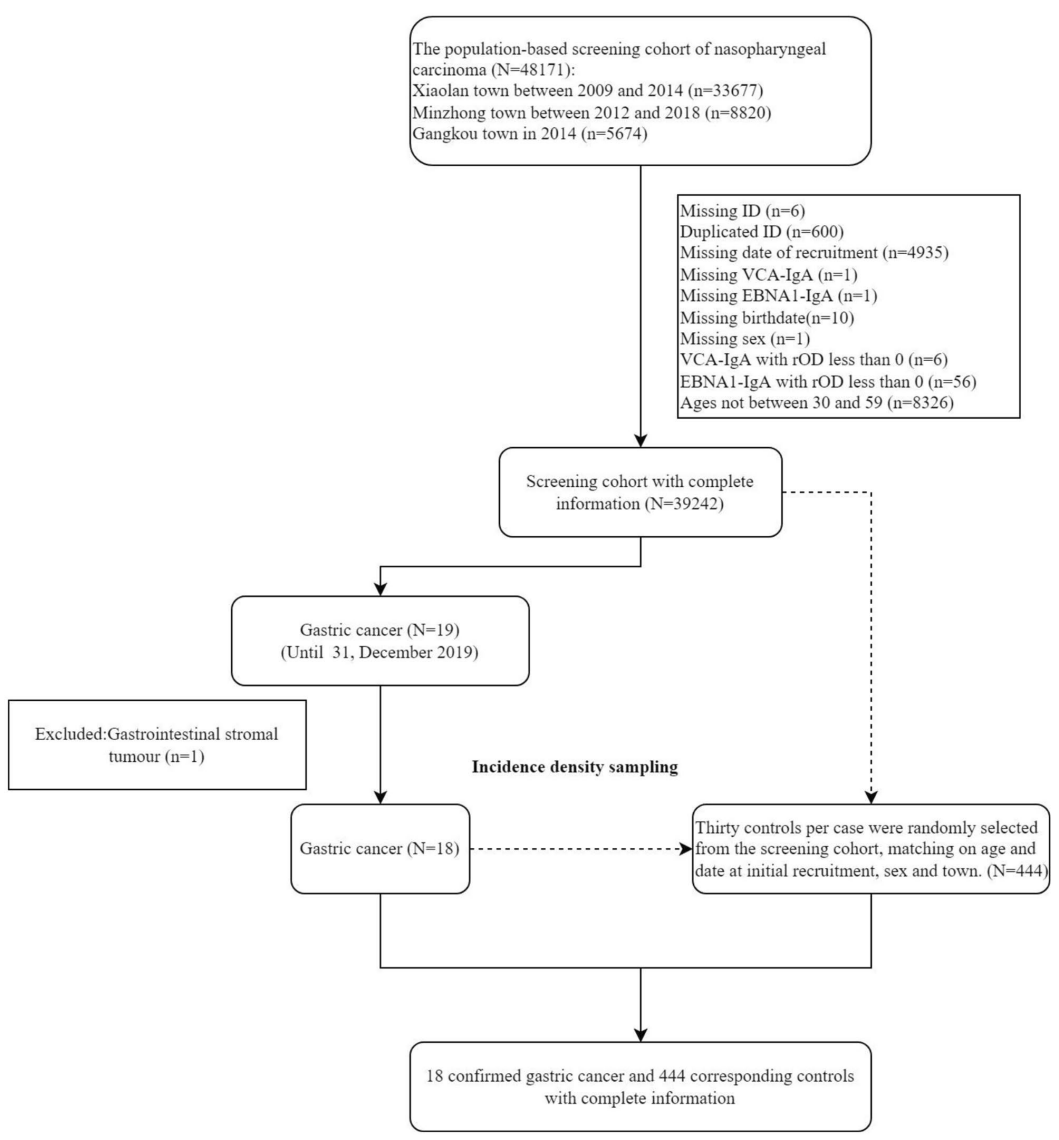
Flow chart of enrollment of study population Note: The exclusion criteria are not mutually exclusive Abbreviations: EBNA1: Epstein-Barr nuclear antigen 1; VCA: Viral capsid antigen; IgA: Immunoglobulin A; rOD: relative optical density

We included 18 pathologically confirmed incident gastric cancer cases and 444 controls (Fig. 1). Among the cases, 15 (83.3%) were males, and 15 (83.3%) were aged 50 to 59 years at recruitment to the screening cohort. The positive proportions of EBNA1-IgA and VCA-IgA were 11.1% (2/18) and 11.1% (2/18), respectively. Among the 444 matched controls, 22 (5.0%) were EBNA1-IgA positive and 26 (5.9%) were VCA-IgA positive, respectively (Table 1). The serological high-risk score by combining two antibodies constituted 16.7% (3/18) in gastric cancer cases and 2.9% (13/444) in controls. The median time interval between serum collection and gastric cancer diagnosis was 3.04 (0.04, 7.59) years (Figure S1).

**Table 1:** Baseline characteristics in gastric cancer cases and controls

Characteristics	Gastric cancer (N = 18)	Controls (N = 444)	Total (N = 462)	P^†^
Sex, N (%)				1.000
Female	3 (16.7%)	90 (20.3%)	93 (20.1%)	
Male	15 (83.3%)	354 (79.7%)	369 (79.9%)	
Age at recruitment, N (%)				1.000
30 ~ 39	1 (5.6%)	30 (6.8%)	31 (6.7%)	
40 ~ 49	2 (11.1%)	60 (13.5%)	62 (13.4%)	
50 ~ 59	15 (83.3%)	354 (79.7%)	369 (79.9%)	
Town, N (%)				0.655
Gangkou	2 (11.1%)	40 (9.0%)	42 (9.1%)	
Minzhong	4 (22.2%)	53 (11.9%)	57 (12.3%)	
Xiaolan	12 (66.7%)	351 (79.1%)	363 (78.6%)	
Tumor histology				
Adenocarcinoma	8 (44.4%)			
Squamous cell carcinoma	2 (11.1%)			
Unknown	8 (44.4%)			
Tumor location				
Cardia	1 (5.6%)			
Non-cardia	6 (33.3%)			
Unknown	11 (61.1%)			
EBNA1-IgA, N (%)				0.399
Negative	16 (88.9%)	422 (95.0%)	438 (94.8%)	
Positive	2 (11.1%)	22 (5.0%)	24 (5.2%)	
EBNA-IgA, rOD				0.984
Median (Min, Max)	0.18 (0.02, 5.49)	0.19 (0.01, 2.57)	0.19 (0.01, 5.49)	
VCA-IgA, N (%)				0.530
Negative	16 (88.9%)	418 (94.1%)	434 (93.9%)	
Positive	2 (11.1%)	26 (5.9%)	28 (6.1%)	
VCA-IgA, rOD				0.137
Median (Min, Max)	0.40 (0.14, 3.86)	0.30 (0.03, 3.05)	0.31 (0.03, 3.86)	
Serological risk (combination of VCA-IgA and EBNA-IgA), N (%)				0.031
Medium/Low	15 (83.3%)	431 (97.1%)	446 (96.5%)	
High	3 (16.7%)	13 (2.9%)	16 (3.5%)	
VCA-IgA and EBNA-IgA, N (%)				0.422
Double negative	14 (77.8%)	398 (89.6%)	412 (89.2%)	
Single positive	4 (22.2%)	44 (9.9%)	48 (10.4%)	
Double positive	0 (0%)	2 (0.5%)	2 (0.4%)	

^†^P values for the difference between case and control across numeric variables were derived by Kruskal-Wallis test while categorical variables by Fisher exact tests.

Abbreviations: rOD, relative optical density.

### Associations of anti-EBV antibodies with risk of gastric cancer

**Fig. 2:**
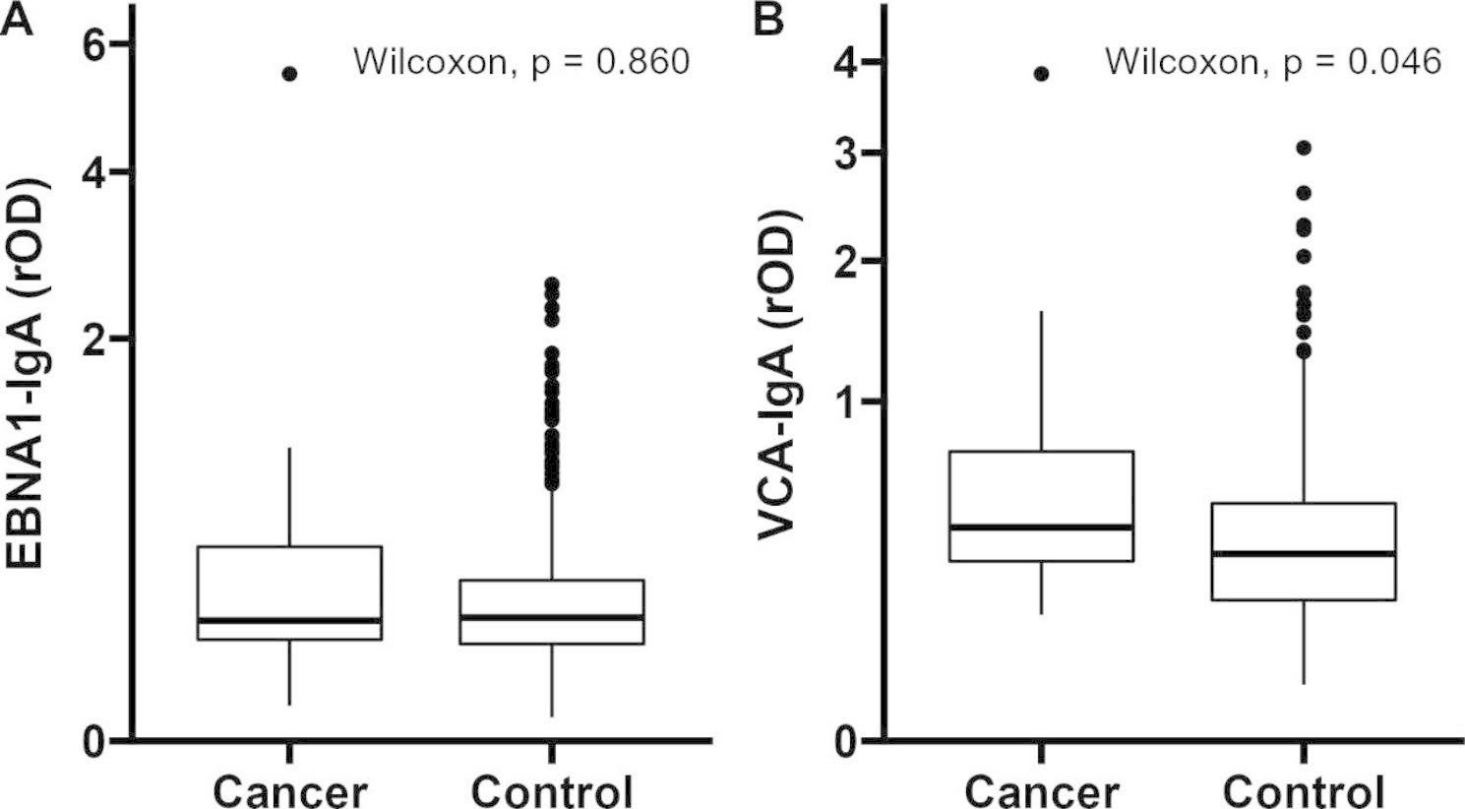
Boxplots of the distribution of rOD of EBNA1-IgA (Panel A) and VCA-IgA (Panel B) in gastric cancer cases and controls. Differences were compared by Wilcoxon rank-sum test Abbreviations: EBNA1: Epstein-Barr nuclear antigen 1; VCA: Viral capsid antigen; IgA: Immunoglobulin A; rOD: relative optical density

The distribution of the rOD of EBNA1-IgA did not differ significantly between cases and controls (*p* = 0.860) (Fig. 2A), whereas that of VCA-IgA was statistically higher among gastric cancer cases than controls (*p* = 0.046) (Fig. 2B).

Although categorical seropositivity for EBNA1-IgA and VCA-IgA was not significantly associated with gastric cancer risk, continuous rOD for either EBNA1-IgA or VCA-IgA was associated with an increased risk of gastric cancer, with adjusted OR of 1.99 (95%CI: 1.07, 3.70) and 2.64 (95%CI: 1.33, 5.23), respectively (Table 2).

**Table 2:** Associations of anti-EBV antibodies with Odds ratio (OR) of gastric cancer risk

	Cases	Controls	Crude OR (95% CI)	Adjusted OR (95% CI)^†^	Adjusted OR (95% CI)^††^
EBNA1-IgA					
Negative	16 (88.9%)	422 (95.0%)	ref	ref	ref
Positive	2 (11.1%)	22 (5.0%)	2.59 (0.55,12.11)	2.59 (0.55,12.13)	2.59 (0.55,12.13)
EBNA1-IgA, rOD			1.98 (1.07,3.68)	1.99 (1.07,3.70)	1.99 (1.07,3.70)
VCA-IgA					
Negative	16 (88.9%)	418 (94.1%)	ref	ref	ref
Positive	2 (11.1%)	26 (5.9%)	2.16 (0.47,9.82)	2.15 (0.47,9.95)	2.15 (0.47,9.95)
VCA-IgA, rOD			2.63 (1.33,5.20)	2.64 (1.33,5.23)	2.64 (1.33,5.23)
Serological risk (combination of VCA-IgA and EBNA-IgA)					
Medium/Low	15 (83.3%)	431 (97.1%)	ref	ref	ref
High	3 (16.7%)	13 (2.9%)	6.49 (1.68,25.00)	6.53 (1.69,25.26)	6.53 (1.69,25.26)

Abbreviations: EBV, Epstein-Barr virus; rOD, relative optical density; EBNA1: Epstein–Barr nuclear antigen 1; VCA: capsid antigen; IgA: Immunoglobulin A.

^†^OR was adjusted by age (numeric) at initial recruitment.

^††^OR was adjusted by age (numeric) at initial recruitment, sex, initial screening date, and residential town.

Participants in the serologically defined high-risk group were at strikingly higher risk of gastric cancer compared with those in the medium- or low-risk group (OR = 6.53, 95% CI: 1.69, 25.26).

**Fig. 3:**
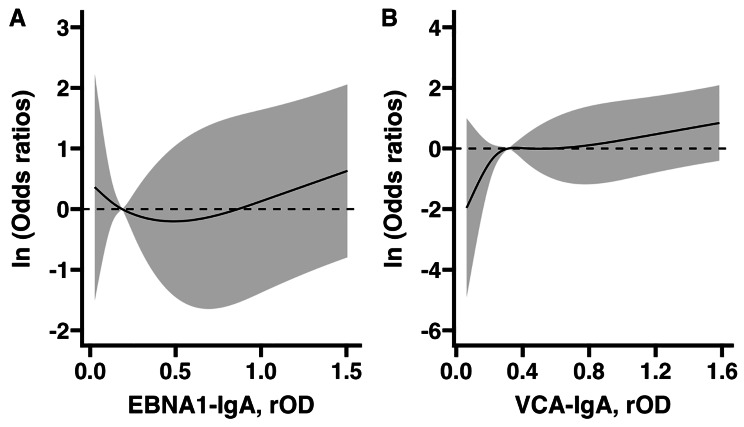
Non-linear associations between rOD of EBNA1-IgA (Panel A) and VCA-IgA (Panel B) and gastric cancer risk Odds ratios were adjusted for continuous age at initial screening Abbreviations: OR, odds ratio; EBNA1: Epstein-Barr nuclear antigen 1; VCA: Viral capsid antigen; IgA: Immunoglobulin A; rOD: relative optical density

The non-linear association between rOD of EBNA1-IgA and VCA-IgA were presented in Fig. 3. ORs of rising rOD VCA-IgA for gastric cancer were consistently increasing (Fig. 3B), while the ORs for EBNA1-IgA showed a slightly short decreasing trend before 0.25, followed by an increasing trend (Fig. 3A).

### Sensitivity analysis

1) Based on repeated incidence density sampling, the average adjusted ORs based on 30 sampling sets were 1.96 (95% CI: 1.81, 2.17) for rOD of EBNA1-IgA and 2.29 (95%CI: 1.92, 3.02) for VCA-IgA, closely matching the results of the primary analysis (Figure S2).

2) The point estimates of ORs for continuous variables were similar, although with wider 95% CIs, after restriction to cases (and their matched controls) with serum samples collected at least two years before gastric cancer diagnosis (Tables S1 and S2, Figure S3).

3) Single positive of EBNA1-IgA and VCA-IgA had much higher odds compared to double negative group without significance (adjusted OR = 2.81, 95%CI: 0.87, 9.13) (Table S3).

4) Non-parametric bootstrapping technique did not show significant associations of time to GC onset and VCA-IgA/EBNA1-IgA magnitude (Figure S4).

## Discussion

Based on this population-based, nested case-control study in southern China, we found that higher pre-diagnosis serological EBNA1-IgA and VCA-IgA are associated with a doubling in risk of subsequent gastric cancer. Moreover, a combination of EBNA1-IgA and VCA-IgA indicating high serological risk was associated with a nearly seven-fold elevation in subsequent gastric cancer risk. This association persisted after restriction to cases with samples collected at least two years before diagnosis.

During primary infection with EBV, which typically occurs in early childhood, EBV crosses the epithelial cells, replicates in B cells, and then invades and establishes latent infection in epithelial cells [12]. In gastric cancer, EBV is an epithelial infection, presumably occurring via the ephrin A2 receptor [13], although the entry mechanism is not conclusive in gastric cancer [14]. EBNA1 is a protein product found across all EBV infection stages from entry to replication [12]. EBNA1 attaches to the viral genome at the origin of replication, initiates and mediates replication, and promotes the division and anchors of the episome for distribution of viral genomes to offspring cells during memory B-cell division [15]. In addition, EBNA1 triggers the transcription of other latent genes essential to cell immortalization. VCA is a structural protein, produced in the late phase of EBV lytic replication, that forms the viral capsid [12]. Upon initial exposure to the Epstein-Barr virus (EBV) structural protein, B cells are activated, and a portion of these B cells differentiate into plasma cells, which produce IgM within four to six weeks. Later on, IgG is produced and persists for the rest of an individual’s life. The timing of IgM, IgG, and IgA production, as well as the expression of EBV antigens during its life cycle, can help diagnose the stage of the infection [16, 17]. For instance, the presence of VCA-IgM indicates the early phase of de novo acute infection, VCA-IgA indicates the early phase of reactivation, and EBNA1-IgA indicates the lytic phase of recent infection [18].

In serum, IgA binds to Fc factor on immune effector cells, thereby triggering an inflammatory reaction to eliminate the virus [19]. Usually, antibodies to EBNA1 are not detected in acute primary infection, but instead develop two to four months afterwards and persist for a lifetime [20]. Positive VCA-IgA indicates previous repeated infection or frequent reactivation of latent EBV infection in B cells [20].

Our results are consistent with some previously reported findings by Shinkura et al. [21] and Aragonés et al. [22]. In the first study, a retrospective case-control study of 123 gastric cancer cases (64 EBV-positive and 59 EBV-negative) and 73 controls in Japan, EBV-positive gastric cancer had higher seropositive rate of VCA-IgA than in EBV-negative gastric cancer. Additionally, the geometric mean titer of VCA-IgG measured at the time of diagnosis in EBV-negative carcinoma cases was higher than that of healthy controls (*P* = 0.028) [21]. Since VCA-IgG indicated the past infection, the EBV infection prevalence might be different between gastric cancer and healthy controls. In the more recent study, a retrospective population-based case-control study in Spain that included 264 gastric cancer cases and 2,071 controls, increasing antibody reactivity against EBNA-1 and VCA, but not the EBV proteins early antigen diffuse and BZLF1-encoded replication activator (ZEBRA) was associated with a higher risk of gastric cancer. Neither of these studies measured anti-EBV antibodies in pre-diagnostic serum samples. Two other retrospective case-control studies in Japan [23] and northern China [24] did not find positive associations between VCA-IgA and gastric cancer risk. The latter study, in fact, found an inverse association based on serum samples collected two years after diagnosis, when IgA may instead be regarded as an indicator of strength of the immune response.

A prospective nested case-control study [25] of 1,447 gastric cancer cases and 1,797 controls in Shanghai and Japan found no association between pre-diagnosis antibodies against VCA, EA, EBNA, or ZEBRA and risk of gastric cancer. We postulate that the difference in findings between this study and ours may be due to two explanations. First, Varga et al. (2018) analysed associations with combinations of IgA, IgG, and IgM antibodies against EBV. IgM indicates recent EBV infection, while IgG indicates past EBV infection and is found in a large proportion of adults worldwide [26]. Thus, combining these three antibody isotypes could have diluted associations towards the null. Second, geographic and underlying genetic variation, particularly related to the risk of NPC, may have contributed to the difference in findings. Zhongshan is an area with a high incidence of NPC, a disease that is etiologically related to EBV whereas the study by Varga et al. [25] was performed in regions at low risk of NPC. Thus, underlying population differences in susceptibility to EBV-related diseases may influence the relationship between host antibody response to EBV and risk of gastric cancer though the association is much lower compared to the strong association with high risk of NPC [16].

Our results showing that an antibody pattern signifying high risk of NPC is also associated with higher risk of gastric cancer, may suggest a shared susceptibility to EBV-mediated epithelial malignancies. Another plausible explanation is that the humoral immune response to EBV might not be confined to EBV-related cancers [27]. That is, although increasing anti-EBV antibodies years before the onset of malignancy may indicate an oncogenic role of EBV specifically, the alteration of antibodies could alternatively reflect a non-specific activation or impairment of immunity in general, which might favor cancer development. These hypotheses could be tested by measuring other non-specific indicators of immune status prior to diagnosis and/or by testing whether an altered anti-EBV antibody response is associated specifically with risk of EBV-positive but not EBV-negative gastric cancer.

There are several strengths of our study. First, the measurement of anti-EBV antibodies in pre-diagnosis serum samples minimizes the potential for reverse causation. Second, to our knowledge, ours is the first study of this kind to evaluate associations between EBNA1-IgA and VCA-IgA in southern China, an endemic area for another EBV-related malignancy NPC. Third, the nesting of this study in a population-based cancer-screening cohort enabled close matching of cases and controls based on demographic characteristics and temporal factors to enable control for confounding, as well as incidence density sampling to enable interpretation of the ORs as incidence rate ratios [28].

We acknowledge the limitations of our study. First, with only 18 gastric cancer cases, statistical results were imprecise. However, our sensitivity analysis based on repeated incidence density sampling indicated that our findings were robust. Second, we used rOD of antibodies as a proxy for titer level, which was not available from our antibody testing method. However, ELISA methods for measuring EBNA1-IgA and VCA-IgA are now well established and are flexible for implementation in real-world screening programs. Third, the incidence of gastric cancer in the screening cohort was lower than that in Zhongshan City overall [3], suggesting that our screening cohort may have lower risk of gastric cancer due to differences in lifestyle and behavioural risk factors (e.g., smoking and diet). These differences would reduce the generalizability of our findings, as may be the setting in southern China, a region endemic for NPC. Fourth, we lacked information on tumour EBV status, which is necessary to determine whether the association with anti-EBV antibodies is specific to EBV-positive gastric cancer, as well as *H. pylori* infection status, which may also affect associations with EBV. Moreover, the latter information could have enabled us to examine whether the combination of elevated antibodies against EBV and *H. pylori* would be even more strongly associated with gastric cancer risk. Finally, we acknowledged the limitation that we have uncontrolled potential residual confounders, i.e., smoking, alcohol consumption, obesity, dietary factors, and family history of gastric cancer. While we have attempted to control for these factors by adjusting for known confounding variables, the possibility of residual confounding cannot be completely ruled out. Therefore, caution is warranted when interpreting our results and future studies may benefit from additional efforts to measure and adjust for these potential confounders.

In summary, our work reveals a positive association between pre-diagnosis EBNA1-IgA and VCA-IgA and risk of subsequent gastric cancer in southern China. The association was especially strong based on a combination of the two antibodies. We thus postulate that EBNA1-IgA and VCA-IgA may be potential markers for early detection or screening of gastric cancer. Additional research is needed to understand how this association may vary by tumor EBV status, how the anti-EBV antibody response may interact with *H. pylori* infection and other possible gastric cancer markers, such as pepsinogen, and the underlying biological mechanism of EBV oncogenesis in gastric cancer and other malignancies.

## Electronic supplementary material

Below is the link to the electronic supplementary material.

Supplementary Material 1

## Data Availability

The datasets generated and/or analysed during the current study are not publicly available due to the policy of the Institute but are available from the corresponding author upon reasonable request.

## Abbreviations

EBV: Epstein-Barr virus
EBNA1: Epstein–Barr nuclear antigen 1
IgA: Immunoglobulin A
NPC: Nasopharyngeal carcinoma
rOD: Relative optical density
VCA: Viral capsid antigen
