# Supplementary material for: EBV antibody and gastric cancer risk: a population-based nested case-control study in southern China
Source: BMC Cancer. 2023 Jun 8;23:521. doi: 10.1186/s12885-023-10994-0 (PMC10251656; doi:10.1186/s12885-023-10994-0)
Supplement: Supplementary file 1 — Supplementary Material 1 [file 12885_2023_10994_MOESM1_ESM.docx]

**Supplementary**

**EBV antibody and gastric cancer risk: a population-based nested case-control study in southern China**

Yun Du^1, 2, ǂ^, Xia Yu^1, ǂ^, Ellen T. Chang^3, ǂ^, Li Yin^2^, Shifeng Lian^4^, Biaohua Wu^1^, Fugui Li^1^, Zhiheng Liang^1^, Yumei Zeng^5^, Bing Chu^5^, Kuangrong Wei^1^, Jiyun Zhan^6^, Xuejun Liang^6^, Weimin Ye^2, §^, Mingfang Ji^1, §^

1, Cancer Research Institute of Zhongshan City, Zhongshan City People’s Hospital, Zhongshan 528400, People’s Republic of China;

2, Department of Medical Epidemiology and Biostatistics, Karolinska Institutet, Stockholm 17177, Sweden;

3, Exponent, Inc., Center for Health Sciences, Menlo Park, 94025, CA, United States;

4, Unit of Integrative Epidemiology, Institute of Environmental Medicine, Karolinska Institutet, Stockholm 17177, Sweden;

5, Department of Pathology, Zhongshan City People’s Hospital, Zhongshan 528400, People’s Republic of China;

6, Xiaolan Public Health Service Center, Zhongshan 528400, People’s Republic of China.

Correspondence to:

Mingfang Ji (Tel: 86 760 89880417; Email: [jmftbh@sina.com](mailto:jmftbh@sina.com)) and Weimin Ye (Tel: +46-(0)8-524 861 84; Email: [weimin.ye@ki.se](mailto:weimin.ye@ki.se))

^ǂ^ Yun Du, Xia Yu and Ellen T. Chang contributed equally.

^§^ Mingfang Ji and Weimin Ye contributed equally.

# Figure S1


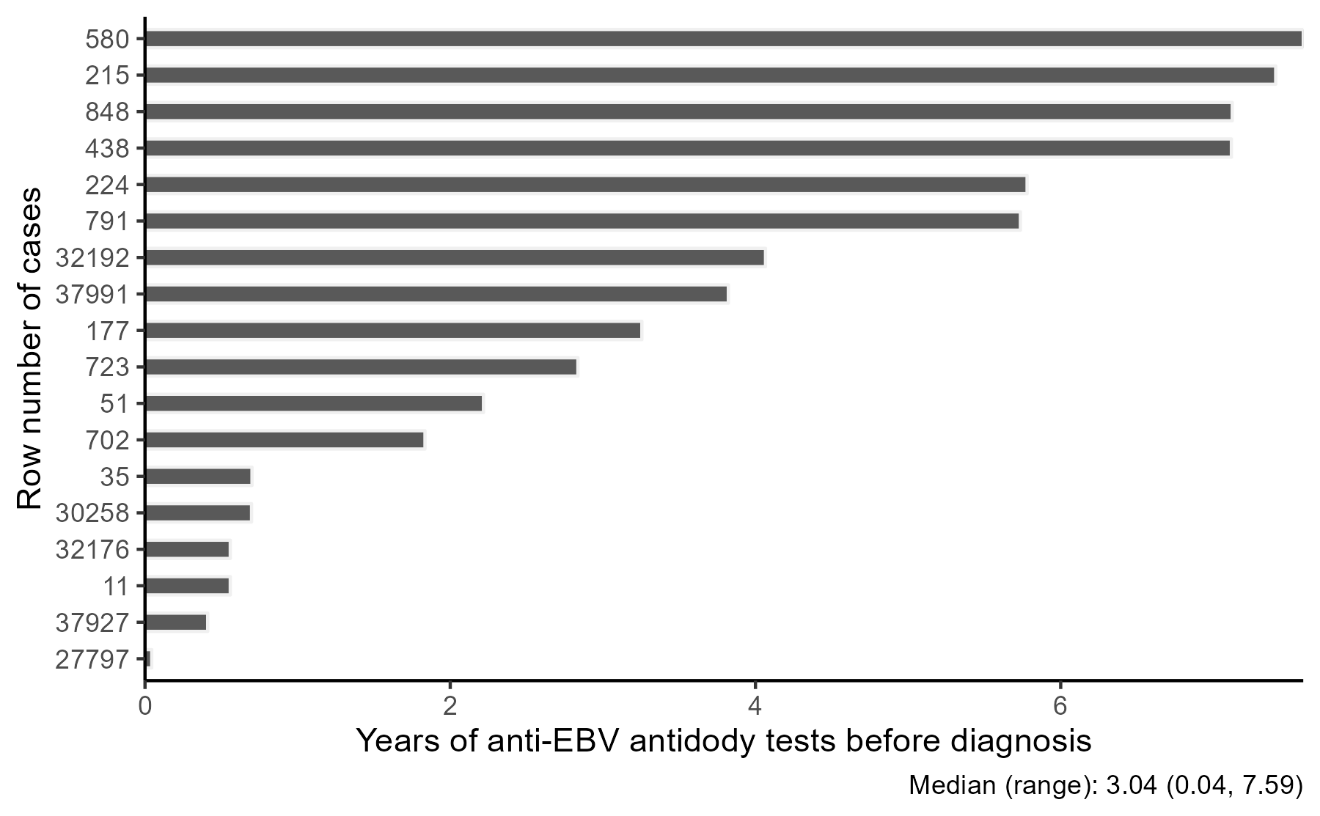


**Figure S1**: Years of anti-EBV antibody tests before cancer diagnosis.

# Figure S2


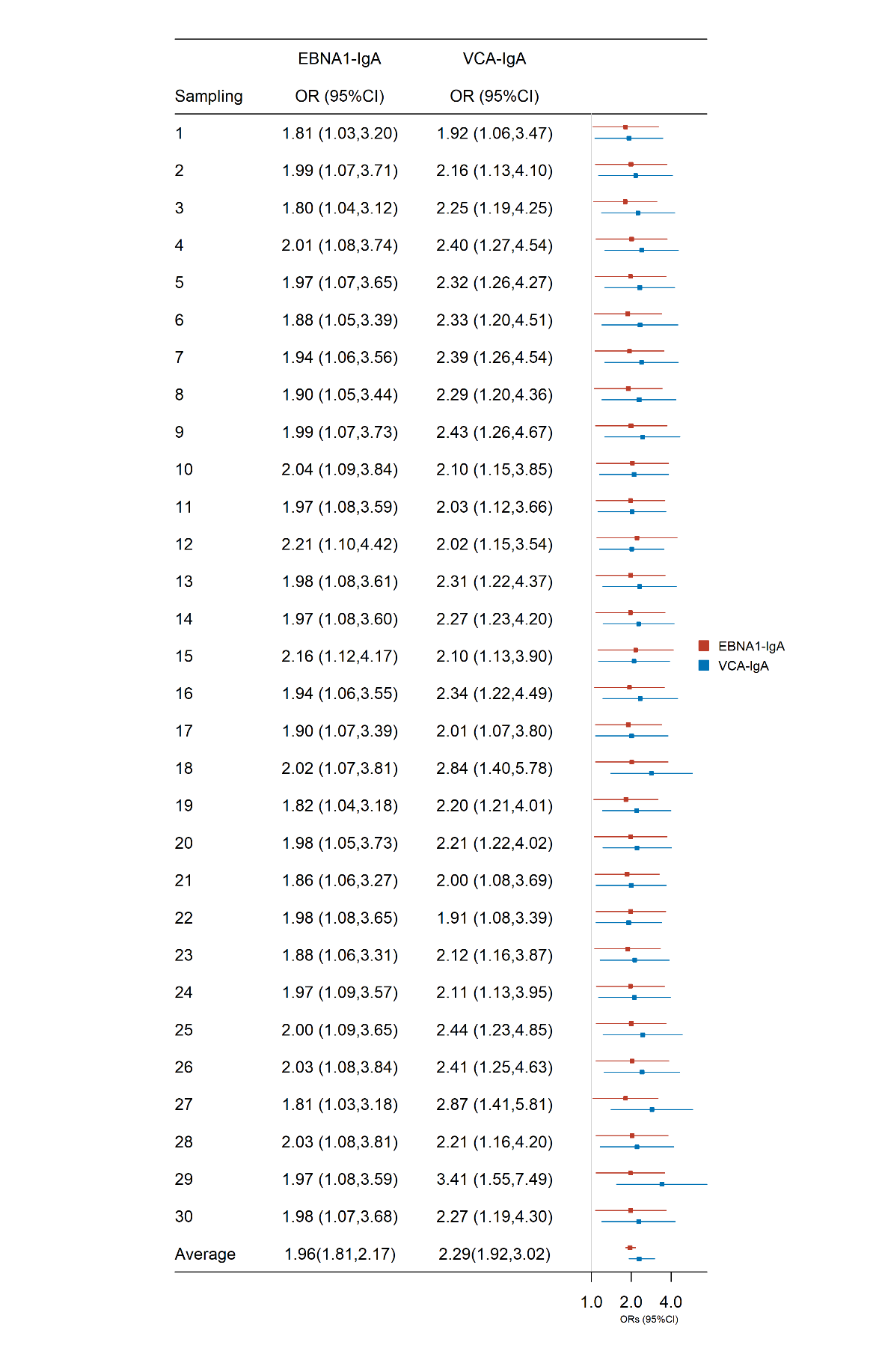


**Figure S2**: Adjusted ORs for EBNA1-IgA and VCA-IgA in association with gastric cancer risk in 30 randomly selected incidence density sampling sets.

Abbreviations: OR, odds ratio; EBNA1: Epstein-Barr nuclear antigen 1; VCA: Viral capsid antigen; IgA: Immunoglobulin A; rOD: relative optical density.

# Figure S3


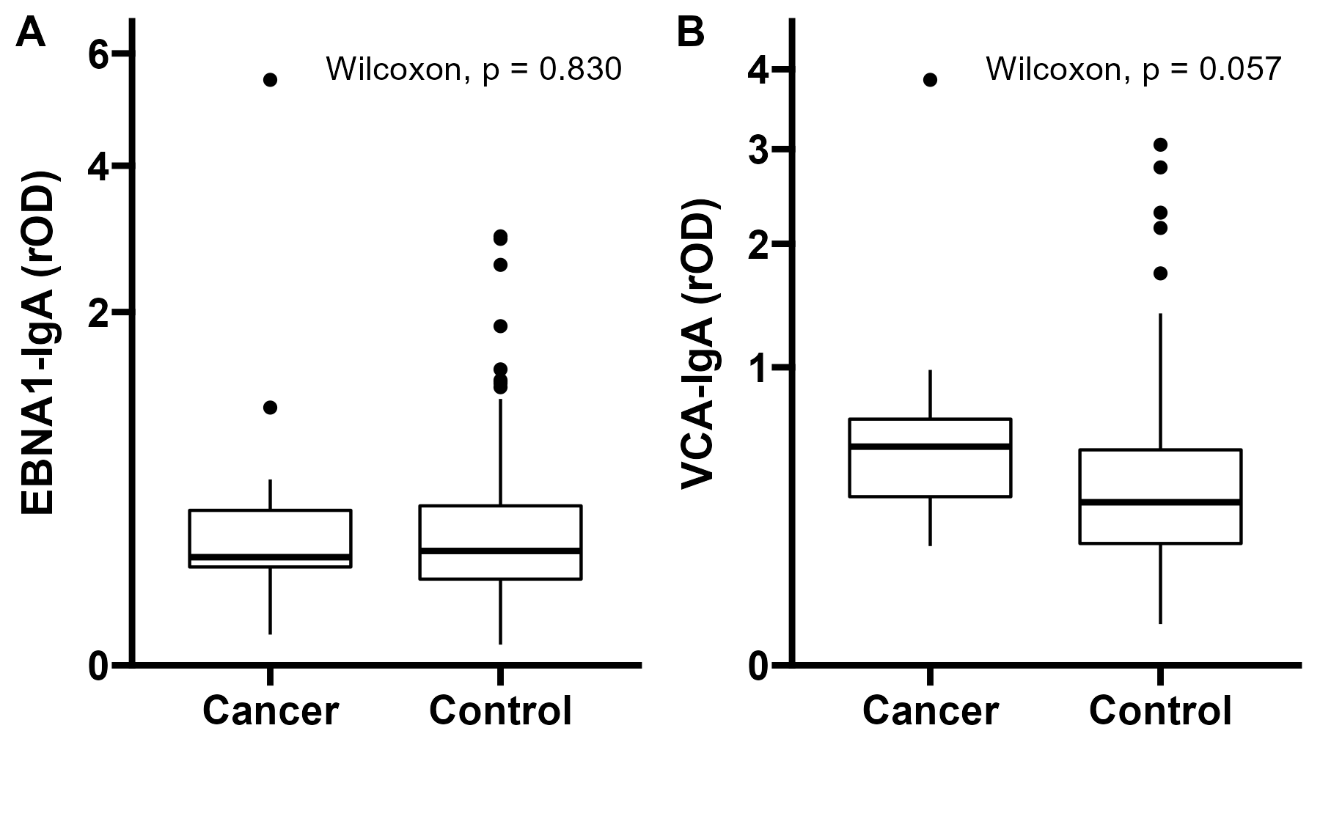


**Figure S3**: Boxplots for rOD of EBNA1-IgA (A) and VCA-IgA (B) in association with gastric cancer risk, restricting to cases with serum samples collected at least two years before gastric cancer diagnosis, along with their matched controls.

Abbreviations: OR, odds ratio; EBNA1: Epstein-Barr nuclear antigen 1; VCA: Viral capsid antigen; IgA: Immunoglobulin A; rOD: relative optical density.

# Figure S4


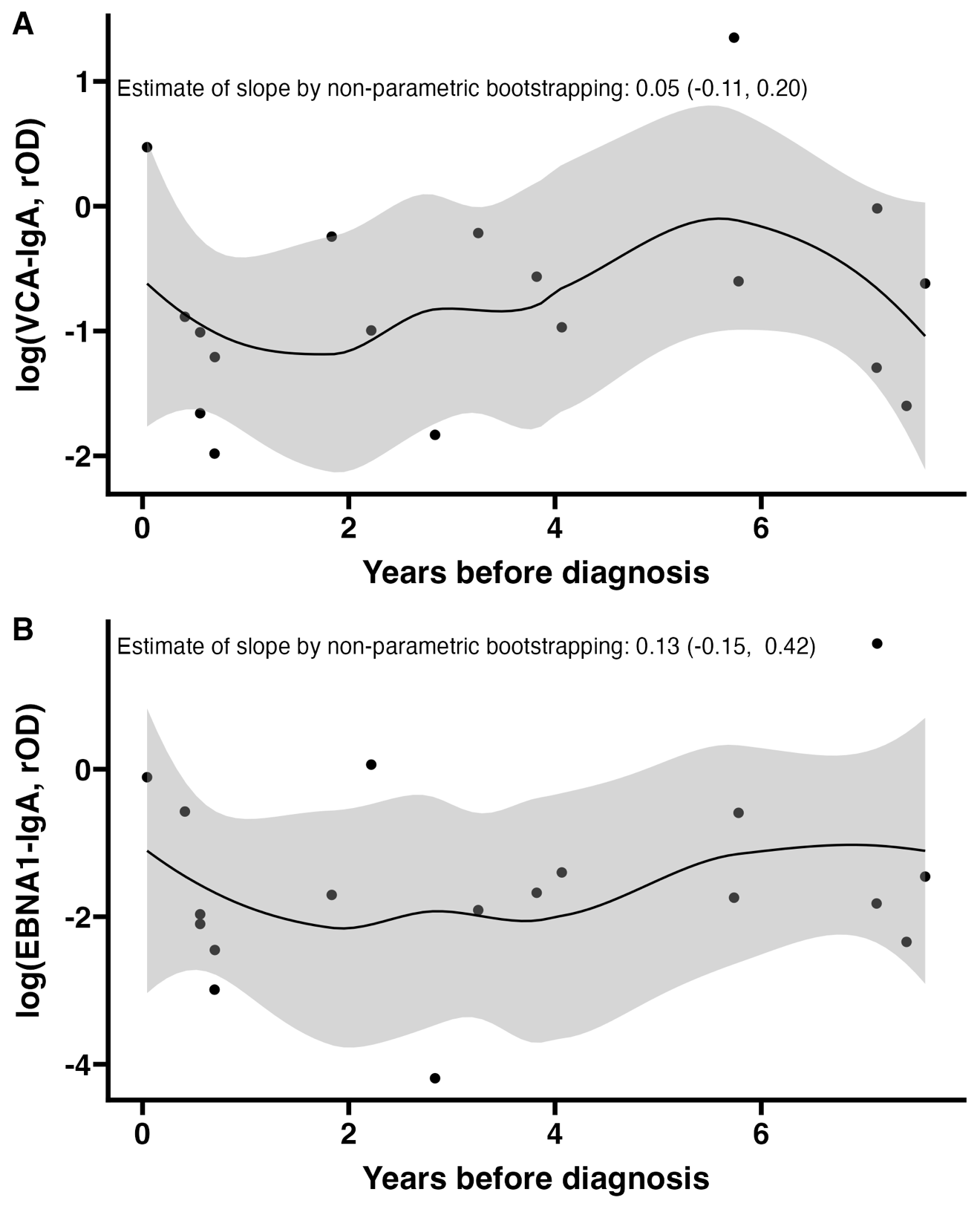


**Figure S4**: Scatter plot of magnitude of VCA-IgA (**Panel A**) and EBNA1-IgA (**Panel B**) along years before gastric cancer diagnosis with estimates (point and 95% CI) by non-parametric bootstrapping.

# Table S1

Table S1. Baseline characteristics of gastric cancer cases and controls^*^

| **Characteristics** | **Cases  (N=11)** | **Controls   (N=289)** | **Total   (N=300)** |
| --- | --- | --- | --- |
| Sex, N (%) |  |  |  |
| Male | 11 (100%) | 289 (100%) | 300 (100%) |
| Age at recruitment, N (%) |  |  |  |
| 30~39 | 1 (9.1%) | 30 (10.4%) | 31 (10.3%) |
| 40~49 | 0 (0%) | 0 (0%) | 0 (0%) |
| 50~59 | 10 (90.9%) | 259 (89.6%) | 269 (89.7%) |
| Town, N (%) |  |  |  |
| Minzhong | 2 (18.2%) | 28 (9.7%) | 30 (10.0%) |
| Xiaolan | 9 (81.8%) | 261 (90.3%) | 270 (90.0%) |
| EBNA1-IgA, N (%) |  |  |  |
| Negative | 9 (81.8%) | 272 (94.1%) | 281 (93.7%) |
| Positive | 2 (18.2%) | 17 (5.9%) | 19 (6.3%) |
| EBNA1-IgA, rOD |  |  |  |
| Median (Min, Max) | 0.19 (0.02, 5.49) | 0.21 (0.01, 2.95) | 0.21 (0.01, 5.49) |
| VCA-IgA, N (%) |  |  |  |
| Negative | 10 (90.9%) | 271 (93.8%) | 281 (93.7%) |
| Positive | 1 (9.1%) | 18 (6.2%) | 19 (6.3%) |
| VCA-IgA, rOD |  |  |  |
| Median (Min, Max) | 0.54 (0.16, 3.86) | 0.30 (0.02, 3.05) | 0.30 (0.02, 3.86) |
| Serological risk score (combination of VCA-IgA and EBNA1-IgA), N (%) |  |  |  |
| Medium/Low | 9 (81.8%) | 283 (97.9%) | 292 (97.3%) |
| High | 2 (18.2%) | 6 (2.1%) | 8 (2.7%) |
| Abbreviations: rOD, relative optical density; EBNA1: Epstein-Barr nuclear antigen 1; VCA: Viral capsid antigen; IgA: Immunoglobulin A.  * Cases were restricted to those with serum samples collected at least two years before gastric cancer diagnosis, along with their matched controls. | | | |

# Table S2

Table S2. Associations of anti-EBV antibodies with gastric cancer risk^*^

|  | **Cases** | **Controls** | **Crude OR (95% CI)** | **Adjusted OR (95% CI)^†^** |
| --- | --- | --- | --- | --- |
| EBNA1-IgA |  |  |  |  |
| Negative | 9 (81.8%) | 272 (94.1%) | ref | ref |
| Positive | 2 (18.2%) | 17 (5.9%) | 4.03 (0.78,20.87) | 4.03 (0.78,20.74) |
| EBNA1-IgA, rOD |  |  | 2.02 (1.06,3.86) | 2.02 (1.05,3.91) |
| VCA-IgA |  |  |  |  |
| Negative | 10 (90.9%) | 271 (93.8%) | ref | ref |
| Positive | 1 (9.1%) | 18 (6.2%) | 1.56 (0.19,12.59) | 1.68 (0.20,13.83) |
| VCA-IgA, rOD |  |  | 2.58 (1.20,5.54) | 2.56 (1.19,5.50) |
| Serological risk score (combination of VCA-IgA and EBNA1-IgA) |  |  |  |  |
| Medium/Low | 9 (81.8%) | 283 (97.9%) | ref | ref |
| High | 2 (18.2%) | 6 (2.1%) | 10.76 (1.89,61.19) | 10.84 (1.90,61.97) |
| Abbreviations: EBV, Epstein-Barr virus; rOD, relative optical density; NPC, nasopharyngeal carcinoma; EBNA1: Epstein-Barr nuclear antigen 1; VCA: Viral capsid antigen; IgA: Immunoglobulin A; OR, odds ratio. | | | | |
| ^†^OR was adjusted for age (continuous) at initial recruitment.  ^*^ Cases were restricted to those with serum samples collected at least two years before gastric cancer diagnosis, along with their matched controls. | | | | |

# Table S3

Table S3. Associations of combined anti-EBV antibodies and gastric cancer risk

|  | **Cases** | **Controls** | **Crude OR (95% CI)** | **Adjusted OR (95% CI)^†^** |
| --- | --- | --- | --- | --- |
| VCA-IgA and EBNA1-IgA |  |  |  |  |
| Double negative | 14 (77.8%) | 398 (89.6%) | ref | ref |
| Single positive | 4 (22.2%) | 44 (9.9%) | 2.80 (0.87,9.05) | 2.81 (0.87,9.13) |
| Double positive | 0 (0%) | 2 (0.5%) | 0.00 (0.00,Inf) | 0.00 (0.00,Inf) |
| Abbreviations: EBV, Epstein-Barr virus; EBNA1: Epstein-Barr nuclear antigen 1; VCA: capsid antigen; IgA: Immunoglobulin A. | | | | |
| ^†^OR was adjusted by age (continuous) at initial recruitment. | | | | |
